# Investigation of Some Metals in Leaves and Leaf Extracts of* Lippia javanica*: Its Daily Intake

**DOI:** 10.1155/2017/1476328

**Published:** 2017-07-11

**Authors:** Kanda Artwell, Ncube France, Kunsamala Florence

**Affiliations:** Department of Environmental Science, Bindura University of Science Education, Private Bag 1020, Bindura, Zimbabwe

## Abstract

Consumption of plant extracts can be a source of essential elements or a route of human exposure to toxicants. Metal concentrations in leaves, leaf brew, and infusion of* L. javanica* collected from five sites were determined by atomic absorption spectrometry after acid and aqueous extraction. Estimated daily intakes of metals in extracts were compared with recommended dietary allowances. Total metal concentrations in leaves varied with sampling sites (*p* < 0.05): Mn > Fe > Cu > Cr > Pb for sites SS2–SS5. The highest metal concentrations in leaves were recorded for SS3 (Cu: 15.32 ± 4.53 and Mn: 734.99 ± 105.49), SS5 (Fe: 210.27 ± 17.17), SS2 (Pb: 3.11 ± 0.21), and SS4 (Cr: 4.40 ± 0.75 mg/kg). Leaf infusion appeared to release higher Cu and Mn concentrations in leaves across sites (Cu: 21.65; Mn: 28.01%) than leaf brew (Cu: 11.95; Mn: 19.74%). Lead was not detected in leaf extracts. Estimated dietary intakes of Cr, Cu, Fe, and Mn were below recommended dietary allowances. A 250 ml cup of leaf infusion contributed 0.30–1.18% Cu and 4.46–13.83% Mn to the recommended dietary allowances of these elements per day. Lead did not pose any potential hazard when consumed in tea beverage made from brew and infusion of leaves of* L. javanica*.

## 1. Introduction

Natural and anthropogenic activities have increased metal concentrations in the environment to unprecedented toxic levels [1–3]. Since plants are capable of taking up metals from soil, the safety, quality, and effectiveness of natural products have become questionable [4]. For example, human dietary exposure to Pb in rice has been reported in previous studies [5, 6]. The consumption of plant tissues may be an important route, not only for essential dietary trace elements but also for human exposure to toxic elements. Various adverse human health effects have been linked to ingestion of toxic metals [7–9]. The likelihood of developing symptoms of disease due to metal toxicity has been attributed to risk factors such as characteristics of the metal (concentration, form, dose, and toxicity) and individual level factors (social, health, behaviour, and physiology) [8, 10, 11].

*Lippia javanica* (Burm F.) Spreng of the family Verbenaceae is a common woody shrub found in different soil types in most parts of central and southern Africa [12, 13], tropical Africa, and central and southern Americas [14]. It grows in open veld, bush, and forest margins [12]. The chemistry, pharmacology, and uses of leaves of* L. javanica* are well documented [13–15]. Apart from its herbal applications, dried leaves of* L. javanica* are used as a tea alternate in routine diet in some parts of South Africa [12] and Zimbabwe. It appears that there is little information available for the concentration of metals in leaves of* L. javanica* and their potential contribution to human diet. There are no recommended quantities or procedures for making tea beverage using dried leaves of* L. javanica* as these vary with households. Information on acceptable levels of metals in leaves or the final brew and infusion extracts and metal contributions in the daily human diet is also not available. Such information is important to avoid deficient or excessive metal intakes.

The human body requires trace elements for healthy growth, development, and proper body functioning [16]. Studying their contribution to the human diet becomes very important because excessive or insufficient intake may cause various nutritional diseases. Chromium potentiates insulin action, influencing carbohydrate, lipid, and protein metabolism [17]. Chromium (III) is an essential element with low toxicity and rare deleterious effects of excessive intake [17]. Chromium (VI) is toxic and has respiratory, cardiovascular, gastrointestinal, hematological, hepatic, and neurological effects [11]. Copper is an essential element for the normal function of more than 30 enzymes, incorporated into metalloenzymes involved in haemoglobin formation, xenobiotic metabolism, carbohydrate metabolism, catecholamine biosynthesis, and antioxidant defence mechanism [18]. Deficiency of Cu in humans may induce anaemia, leukopenia, and osteoporosis [18]. Manganese is an activator and constituent of several enzymes which regulate lipid and carbohydrate metabolism, bone and tissue formation, and skeletal growth [19]. It is considered among the least toxic of trace elements when administered orally and excessive intake of Mn by ingestion is not a problem as the body regulates it homeostatically [17]. Iron is an essential element in haemoglobin, myoglobin, ferredoxins, and several enzymes active in porphyrin synthesis, transport of oxygen, and normal functioning of the immune system [20]. Deficiency in Fe causes anaemia. Infants, children, and pregnant mothers are the most susceptible to anaemia, an Fe deficiency [20]. Lead is a xenobiotic toxicant which can accumulate in body organs (gastrointestinal tract, and kidneys) and the central nervous system leading to poisoning [21–24]. Children are most vulnerable to Pb poisoning due to neurological, neurobehavioural, and developmental effects [22]. Lead has been classified as a category 2B carcinogen (possible carcinogenicity to humans) by the IARC [25] but with inadequate carcinogenicity evidence for humans.

The current study determined (1) total concentrations of metals (Cr, Cu, Fe, Mn, and Pb) in leaves of* L. javanica* by acid digestion and (2) concentrations of elements in brew and infusion extracts. Also, contributions of tea brew and infusion made from leaves of* L. javanica* to dietary intake of metals were determined. The study was premised on the hypotheses that sampling sites influenced metal concentrations in* L. javanica* leaves. The solubility of a metal was assumed to be dependent on the method of extraction and the specific metal.

## 2. Materials and Methods

### 2.1. Sampling, Sample Preparation, and Analysis

Seven sites were purposively selected for sampling leaves of* L. javanica*: along sides of a railway line (SS1), busy road side (SS2), at a municipal solid waste dumpsite (SS3), in an informal light industrial area (SS4), around a sewage outfall (SS5), grassland (SS6), and a natural forest (SS7). Sampling was done in Marondera, Zimbabwe (lat. 018.18527 and long. 031.55193). Healthy green leaves without signs of parasitic herbivory or disease were sampled from three* L. javanica* plants at each site after the flowering stage. The leaves were cleaned with deionised water. They were put into polythene bags and shipped to the laboratory and air-dried for seven days. Three air-dried samples from each site were separately ground (SM-450-C grinder) and sieved (nylon 0.425 mm). Sample preparation and analysis were done following procedures described by Soomro et al. [16] with minor modifications for boiling time and hot water extraction volumes. For the determination of total metal concentration, three powdered samples (2 g each) were weighed into Teflon vessels. A 12 ml concentrated acid mixture (69% HNO_3_ : 70% HClO_4_; 3 : 1 v/v) was used for digestion over a sand bath in a fume hood until it became clear. The cooled digests were filtered into a 100 ml volumetric flask and made to the mark with 5% HNO_3_. For hot water extraction, three similar powdered leaf samples from the same site were each separately added to boiling distilled water (250 ml) for five minutes over a hot plate, cooled, and filtered (Whatman No. 42) under gravity.

To determine the fraction of element extracted by infusion of leaf samples in boiling water, three 2 g leaf samples were separately added to each of a third set of three conical flasks in which 250 ml of boiling distilled water were added. They were allowed to stand for 5 minutes, cooled, filtered under gravity, and evaporated to near dryness. The residues were acid-digested and the final volume adjusted as described above. Ten sample solutions were analysed for their elemental concentrations alongside a reagent blank solution using flame atomic absorption spectrometry. The digestion procedure was validated by using spike-and-recovery experiments for the metals. In this procedure, a laboratory sample was spilt into four replicate aliquots. For the analysis of total metal concentration, a homogenised sample of leaf powder was used. Evaporation residues of leaf brew and infusion were each separately used for validating the digestion procedure. An analyte of known concentration was separately added to three aliquots of each laboratory sample. The four samples (including the unspiked aliquot) were digested and analysed following the general procedure. Element recovery (%) was determined as the difference between the spiked and unspiked samples divided by the spike sample added, expressed as a percentage [27]. The method detection limit (MDL) was determined by running ten method blank solutions and expressing it as three times the mean standard deviation of blank solutions [27].

Concentrations of elements were determined by extrapolation from a calibration graph developed from working standards made by serial dilutions of appropriate stock solutions. The spectrometer (Thermo Scientific model iCE 3000 series) was optimised for element analysis following instructions of the manufacturer (Table 1). Analyte samples and blanks were aspirated into the instrument. Standard solutions were rerun after every batch of 10 samples to check and correct any instrumental drift. Estimated daily intake (EDI) of trace element was determined and compared with recommended dietary allowances (RDA) following procedures described by Brzezicha-Cirocka et al. [28]. This was done because of lack of information of maximum allowable concentrations of trace elements in tea leaves. The mass of leaves of* L. javanica* used to make the beverage brew and infusions (2 g) was of commercial tea bags (Quickbrew™) commonly available in supermarkets. The volume of tea consumed by an individual (250 ml) was of tea cups used in the study area.

### 2.2. Statistical Analysis

Data were subjected to One-Way ANOVA to determine any significant differences in the concentration of metals extracted by three treatments of leaves of* L. javanica* sampled from seven sites. Where differences were significant, pairwise comparisons were conducted using LSD post hoc. Estimated daily intake values for trace elements were expressed as percent fractions of recommended dietary allowances (DRA) values. All analyses performed were considered significant at 95% level of confidence.

### 2.3. Quality Control Procedures

Concentrated acids used were ACS-grade reagents (Merck, Germany). Triplicate samples were used and each was measured three times. Reagent blanks were run alongside laboratory samples. Instrument recalibration for drift was done by stepwise correction [27] using prepared standards after a batch of 10 analyte samples.

## 3. Results and Discussion

### 3.1. Total Concentrations of Elements in Leaf Samples of* L. javanica*

Table 2 shows that element recoveries from spiked samples were from 91.21 ± 1.46 to 102.41 ± 2.20%. The percentage Relative Standard Deviation (% RSD) values were <10%.  Table 3 shows the concentrations of Cr, Cu, Fe, Mn, and Pb in dried leaf samples of* L. javanica* from seven sampling sites. Total concentrations (acid-digested) of Cr and Pb were not detected in leaf samples from sampling sites SS1, SS6, and SS7. Concentrations recorded for Cu, Fe, and Mn at SS6 and SS7 were not significantly different (*p* > 0.05). Highest total concentrations of elements were recorded at sites SS2 (Pb), SS3 (Cu and Mn), SS4 (Cr), and SS5 (Fe) while lowest values were recorded at sites SS6 and SS7 for elements studied (Table 3). Significant differences in total concentrations of elements were observed for Cr (SS1–SS4), Fe and Mn (SS1–SS5), and Pb (SS2–SS5) (*p* < 0.05). Mean total concentrations of trace elements in leaves of* L. javanica* from seven sampling sites decreased in the order: Mn > Fe > Cu > Pb > Cr.

The presence of trace elements in leaves of* Lippia javanica* reveals that the plant can accumulate them. Undetectable amounts of elements in samples from SS6 (grassland) and SS7 (natural forest) may suggest no point sources except for background concentrations and diffuse source contributions such as atmospheric deposition and surface runoff. Chromium was found the highest in leaf samples from the light industrial area (SS4). High concentrations of Cr were reported in plants growing in high Cr containing soils, for example, near ore deposits, Cr-emitting industries, and soils fertilised with sewage sludge [28]. Copper and Mn were the highest in leaf samples collected from the municipal dumpsite (SS3). Common sources of Cu are waste dumps, domestic wastewater, landfills, agricultural use, wood production, and combustion of fuels [20]. Manganese is commonly released into the environment from steel manufacturing, fireworks, dry-cell batteries, fertilisers, and paints [19].

Iron is ubiquitous in the natural environment in elevated concentrations. Its many applications may elevate concentrations in the soil, finally in the plant. Sewage contains many elements whose availability to plants is limited by many factors including complexity in composition. Lead was found to be the highest in leaves sampled along the busy road (SS2). This may not be expected considering that leaded gasoline has been banned [21]. It may be assumed that leaded gasoline is still being used especially in developing countries. According to ATSDR [21] the highest concentrations of Pb were reported in plants growing near Pb mining sites, storage batteries, spoil disposal areas, sewage sludge, and areas affected by automobile traffic.

It appears currently that there are no studies reporting on concentrations of heavy metals in leaves, leaf brew, and infusion extracts of* L. javanica, *save for Mahlangeni et al. [29], South Africa, where the plant is extensively used as herbal infusion and tea alternative. Most related studies were done on tea products of* Camellia sinensis* bought from market places worldwide [16, 30–33]. Mahlangeni et al. [29] showed that* L. javanica* leaves contained site-dependent metal concentrations from 10 sites in the decreasing order: Fe > Mn > Cu > Cr > Pb compared to Mn > Fe > Cu > Pb > Cr from this study. The highest total metal concentrations reported were about 2.6 (Cr), 9.0 (Cu), 800 (Fe), and 1.25 mg/kg (Pb). Observed differences in elemental concentrations for leaf samples in these two studies could be due to factors that influence their uptake such as soil characteristics and properties of elements [21, 23]. Total metal concentrations in leaves of* Camellia sinensis* were observed to increase with increasing age of the plant [31]. However, postflowering stage and increasing distance from a point source of metal were observed to reduce metal concentration in leaves of* Salvia officinalis*, a herbal tea [34].

### 3.2. Proportion of Elements Extracted from Leaves of* L. javanica* by Leaf Brew and Infusion

Table 3 also shows water extractable fractions of elements from leaves of* L. javanica* by leaf brew and infusion over five minutes. Lead was not detected at all sites by the two treatment methods. Chromium was detected only at SS2 and SS4 in leaf brew and at SS2, SS3, and SS4 in leaf infusion in significantly different concentrations (*p* < 0.05). Leaf infusion appeared to extract a greater proportion of Mn and Cu than brewing. The highest proportions of elements in leaf infusions were recorded for sites SS4 (Cr: 22.71; Cu: 28.65), SS3 (Fe: 24.40), and SS5 (Mn: 32.72%). The overall mean extraction of elements by leaf infusion across all sites followed the decreasing trend Mn > Cu > Fe > Cr > Pb. Leaf brew of* L. javanica* had mean element extractions of 14.03 (Cr), 11.95 (Cu), 15.68 (Fe), and 19.74% (Mn). Mahlangeni et al. [29] reported extraction percentages of 37–68.8 (Pb), 23.1–28.7 (Fe), 21.5–48.8 (Cu), and 71.8–93.9% (Cr) in leaf brew after 10 minutes. These values are higher than both extractions from our study. Variations could be explained by differences in boiling time, method of brewing, and brand of tea which were shown to influence the degree of element extraction from the total content [16, 30]. ATSDR [19] considers many forms of Mn as water soluble. This could explain the high proportion of metal release from the leaf into aqueous solution. The solubility of Pb in water is a function of pH, hardness, salinity, and presence of humic material [21]. The observation that Mn and Cu appeared to be released more into the infusion than brew extracts was explained for* Camellia sinensis* in terms of their potential for chelation with tannins and tannic acid which exudes on boiling (brew) such that when chelates precipitate, element content decreases [35]. In separate studies, little or no Pb was detected in leaves, made tea, and infusions from* Camellia sinensis*, common tea [32, 35, 36].* Camellia sinensis* has been shown to release high concentrations of Mn in made tea and its infusions [28, 30, 36].

The general trend on the content of metals in hot water extracts of* Camellia sinensis* reported by Soomro et al. [16] was Mn > Fe > Cr > Cu > Pb. This is slightly different from our findings (Mn > Cu > Fe > Cr > Pb) for leaf infusion of* L. javanica*. In most tea infusion studies, it has been observed that distilled water is used for elemental extraction [30, 32]. However, in most rural communities where plants are exploited for various purposes, water is of marginal quality with various levels of elements. Water chemistry may influence the final concentrations of elements in the tea brew and infusions, consequently the final dietary intake of elements.

### 3.3. Estimated Daily Intake of Trace Elements

Table 4(a) shows the estimated dietary intake of trace elements by consuming of 250 ml tea beverage made from brew extracts of 2 g of dried leaves of* L. javanica* for seven sites. Table 4(b) shows the same information for leaf infusion. These data are shown against recommended dietary allowances of each element for a specified age group.  Table 5 shows the percent contribution of estimated intake of elements from consuming 250 ml of leaf brew and its infusion to the recommended daily dietary allowance of an element. Results suggest that all elements contributed a greater fraction to the RDA from the infusion extract across all age groups and special considerations (pregnancy and lactation) than leaf brew. Leaf extracts from* L. javanica* appear to contribute very little to the daily metal intake with respect to Fe and Cu whose percentages were less than unity, except for the infusion where Cu contributed 1.18% to children (1–3 years). Consuming one cup of* L. javanica* tea brew contributes 4.50–9.75 while the leaf infusion extracts 4.46–13.83% of the dietary Mn allowance per day. This contribution will increase with the number of cupfuls consumed. The similar cupful of extracts would contribute between 0.67–2.72 (from brew) and 1.11–4.55% (infusion) Cr to the RDA. Results suggest that* L. javanica* may have the potential to contribute significantly to the daily intake of Mn and Cr. Values are more pronounced for children who are the most susceptible to elemental toxicity and deficiency effects. Dietary reference standards are important for planning for meals and to educate consumers on deciding what food to eat and how much. Diet assessments assist in food safety which includes regulation, monitoring, education, and counselling [26].

## 4. Conclusions

The concentrations of Cr, Cu, Fe, Mn, and Pb in leaves, leaf brew, and infusion extracts of the tea alternate,* L. javanica* collected from seven sites, were determined. The study showed that the dietary contribution of Cu, Cr, Fe, and Mn from the consumption of tea beverage was higher in the leaf infusion than the brew of* L. javanica*. However, leaf extracts appear to have very little contribution to toxic Pb dietary intake. Estimated daily intakes of studied metals were within recommended dietary intake values. Exposure to Pb through consuming tea beverage made from leaf brew and infusion of* L. javanica* collected from the seven sites did not constitute a health risk to the local population. Infusion leaf extracts of* L. javanica* may be an important source of Cr and Mn for daily dietary requirement of essential metals considering that tea generally forms part of a daily diet for most people. However, long term studies on dietary exposure to toxic metals through tea made from* L. javanica* growing in contaminated environments may be needed to safeguard human health.

## Figures and Tables

**Table 1:** Operational conditions of the atomic absorption spectrometer (FAAS Thermo Scientific model iCE 3,000 series) used for the analysis of some metals in *L. javanica* leaves and aqueous leaf extracts. The flame was air acetylene^a^.

Metal	Wavelength (nm)	Band pass (nm)	Lamp current (mA)	Fuel flow rate (L/min)	Correlation coefficient (*R*^2^)
Cr	357.9	0.5	10.00	4.2	0.9978
Cu	324.8	0.5	7.50	1.1	0.9992
Fe	248.3^*∗*^	0.2	11.25	0.9	0.9988
Mn	279.5^*∗*^	0.2	7.50	1.1	0.9971
Pb	217.0^*∗*^	0.5	7.50	0.9	0.9985

^a^For Cr the flame was N_2_O-C_2_H_2_.

^*∗*^With D_2_ Quadline background correction.

**Table 2:** Mean concentrations (mean ± SD, mg/kg, DW, *n* = 3), element recoveries (%), RSD (%), and LOD for metals in leaf samples of *L. javanica*  (*n* = 7).

Element	Aliquot	Spiked sample	spike	% recovery	% RSD	LOD (mg/kg)
Cu (T)	10.28	11.50 ± 0.80	2.10	92.89 ± 1.12	6.96	0.230
Cu (I)	3.36	4.98 ± 0.35	2.10	91.21 ± 1.46	7.03	0.230
Fe (T)	104.44	126.26 ± 7.23	20.80	102.41 ± 2.20	5.73	1.250
Mn (T)	86.25	96.51 ± 6.34	17.20	94.26 ± 1.80	6.57	1.540
Mn (B)	39.71	52.77 ± 4.27	17.20	92.72 ± 1.39	8.09	0.120
Pb (B)	0.00	2.34 ± 0.11	2.52	92.73 ± 0.23	4.70	0.120
Cr (I)	0.63	1.57 ± 0.06	1.00	96.32 ± 0.62	3.83	0.188

T: total metal concentration, B: leaf brew, and I: leaf infusion.

**Table 3:** The total concentration of five elements (mg/kg, DW) in dried leaves of *L. javanica* sampled from seven different sites and extracted by three treatments. Values are mean ± SD of triplicate site samples measured three times.

Site	Treatment	Statistic	Element concentration (mg/kg)				
			Pb	Fe	Mn	Cu	Cr
SS 1	Acid digestion	$\overset{-}{y}$	ND	86.386 ± 9.057^A^	121.165 ± 26.388^A^	9.854 ± 1.751^A^	ND
	Boiling in water	% of total	—	16.83 ± 2.94	16.98 ± 4.77	12.95 ± 6.09	—
	Infusion in water	% of total	—	20.58 ± 5.31	30.78 ± 15.53	19.85 ± 4.87	—

SS 2	Acid digestion	$\overset{-}{y}$	3.109 ± 0.214^A^	104.433 ± 7.329^B^	205.431 ± 21.138^B^	10.275 ± 0.958^B^	3.559 ± 0.241^A^
	Boiling in water	% of total	—	15.50 ± 0.91	19.34 ± 0.99	14.00 ± 3.35	8.88 ± 0.97
	Infusion in water	% of total	—	21.69 ± 6.00	29.15 ± 2.93	21.50 ± 3.99	13.54 ± 0.62

SS 3	Acid digestion	$\overset{-}{y}$	1.620 ± 0.035^B^	185.194 ± 10.925^C^	734.988 ± 105.490^C^	15.323^BC^	1.877^B^
	Boiling in water	% of total	—	18.15 ± 0.25	21.11 ± 4.39	14.72 ± 1.90	6.94 ± 1.81
	Infusion in water	% of total	—	24.40 ± 2.02	25.40 ± 0.68	21.95 ± 6.43	12.52 ± 2.66

SS 4	Acid digestion	$\overset{-}{y}$	2.086 ± 0.581^C^	147.269 ± 8.779^D^	287.255 ± 37.604^D^	14.787 ± 2.570^C^	4.404 ± 0.746^C^
	Boiling in water	% of total	—	13.39 ± 1.97	14.70 ± 0.33	12.95 ± 3.45	10.35 ± 2.39
	Infusion in water	% of total	—	15.82 ± 3.66	26.66 ± 9.79	28.65 ± 2.26	14.19 ± 3.24

SS 5	Acid digestion	$\overset{-}{y}$	1.851 ± 0.070^D^	210.226 ± 17.167^E^	554.111 ± 67.406^E^	12.662 ± 3.337^AB^	1.168 ± 0.282^D^
	Boiling in water	% of total	—	17.68 ± 2.56	23.81 ± 1.29	10.67 ± 4.97	—
	Infusion in water	% of total	—	22.99 ± 0.29	32.72 ± 8.60	17.18 ± 3.88	—

SS 6	Acid digestion	$\overset{-}{y}$	ND	37.313 ± 4.988^F^	86.248 ± 13.277^A^	3.816 ± 1.184^D^	ND
	Boiling in water	% of total	—	9.04 ± 1.58	12.49 ± 3.26	3.17 ± 1.15	—
	Infusion in water	% of total	—	11.68 ± 3.64	24.73 ± 7.37	13.23 ± 3.42	—

SS 7	Acid digestion	$\overset{-}{y}$	ND	42.197 ± 6.506^F^	85.736 ± 25.116^A^	3.733 ± 0.590^D^	ND
	Boiling in water	% of total	—	6.83 ± 0.27	10.62 ± 4.36	3.32 ± 1.60	—
	Infusion in water	% of total	—	8.33 ± 2.66	21.19 ± 6.27	21.59 ± 5.34	—

Overall	Acid digestion	$\overset{-}{y}$	2.176	116.153	296.419	10.064	2.752
	Boiling in water	% of total	—	15.68 ± 4.46	19.74 ± 5.15	11.95 ± 5.63	8.72 ± 1.71
	Infusion in water	% of total	—	20.31 ± 6.56	28.01 ± 8.20	21.65 ± 5.33	13.42 ± 0.84

ND: not detected *(below limit of detection)*; dash (—): not calculated.

Treatment means with different superscripts A and Bor AB and C within the same column for a specific element are significantly different (LSD post hoc, *p* < 0.05).

SS 1: along sides of a railway line; SS 2: along sides of a busy road; SS 3: municipal dumpsite; SS 4: around a light industry; SS 5: around a sewage outfall; SS 6: grassland; and SS 7: natural forest.

**Table:** (a) Recommended dietary allowances (RDA, mg/day/person) and estimated dietary intake (EDI, mg/day/person; 250 ml beverage) of trace elements for the consumption of leaf brew extracts of *L. javanica *(2 g, 5 minutes) collected from seven sampling sites of Marondera, Zimbabwe

Metal	RDA for a life stage group (years)								Mean metal concentration at sampling site ($\overset{-}{y}$ ± SD) mg/250 ml							
	Children		Males		Females		Pregnancy	Lactation	SS1	SS2	SS3	SS4	SS5	SS6	SS7	Overall
	1–3	4–8	19–50	>51	19–50	>51	19–50	19–50								
^*∗*^Cr	0.011	0.015	0.035	0.030	0.025	0.020	0.030	0.045	ND —	0.006 ± 0.00007	0.0003 ± 0.00003	0.0009 ± 0.0001	ND —	ND —	ND —	0.0003 ± 0.0003
Cu	0.34 (1.00)	0.44 (1.00)	0.90 (10.00)	0.90 (10.00)	0.90 (10.00)	0.90 (10.00)	1.00 (10.00)	1.30 (10.00)	0.003 ± 0.0007	0.003 ± 0.0004	0.005 ± 0.001	0.004 ± 0.0003	0.003 ± 0.0004	0.0002 ± 0.00007	0.0003 ± 0.00008	0.002 ± 0.002
Fe	7.00 (40.00)	10.00 (40.00)	8.00 (45.00)	8.00 (45.00)	18.00 (45.00)	8.00 (45.00)	27.00 (45.00)	9.00 (45.00)	0.029 ± 0.002	0.032 ± 0.004	0.067 ± 0.005	0.039 ± 0.004	0.074 ± 0.008	0.007 ± 0.0009	0.006 ± 0.0007	0.0364 ± 0.026
^*∗*^Mn	1.20 (2.00)	1.50 (3.00)	2.30 (11.00)	2.30 (11.00)	1.80 (11.00)	1.80 (11.00)	2.00 (11.00)	2.60 (11.00)	0.041 ± 0.006	0.079 ± 0.005	0.310 ± 0.022	0.084 ± 0.010	0.264 ± 0.020	0.022 ± 0.003	0.018 ± 0.003	0.117 ± 0.114

Adapted and modified from Ross et al. [26].

^*∗*^Adequate intake.

Values in brackets: tolerable upper limit.

**Table:** (b) Recommended dietary allowances (RDA, mg/day/person) and estimated dietary intake (EDI, mg/day/person; 250 ml beverage) of trace elements for the consumption of leaf infusion extracts of *L. javanica *(2 g, 5 minutes) collected from seven sampling sites of Marondera, Zimbabwe.

Metal	Life stage group (years)								Mean metal concentration at sampling site ($\overset{-}{y}$ ± SD) mg/250 ml							
	Children		Males		Females		Pregnancy	Lactation	SS1	SS2	SS3	SS4	SS5	SS6	SS7	Overall
	1–3	4–8	19–50	>51	19–50	>51	19–50	19–50								
^*∗*^Cr	0.011	0.015	0.035	0.030	0.025	0.020	0.030	0.045	ND —	0.001 ± 0.00007	0.0005 ± 0.00004	0.001 ± 0.0002	ND —	ND —	ND —	0.0005 ± 0.0005
Cu	0.34 (1.00)	0.44 (1.00)	0.90 (10.00)	0.90 (10.00)	0.90 (10.00)	0.90 (10.00)	1.00 (10.00)	1.30 (10.00)	0.004 ± 0.0005	0.004 ± 0.0005	0.007 ± 0.003	0.008 ± 0.0009	0.004 ± 0.0004	0.001 ± 0.00006	0.002 ± 0.0002	0.004 ± 0.003
Fe	7.00 (40.00)	10.00 (40.00)	8.00 (45.00)	8.00 (45.00)	18.00 (45.00)	8.00 (45.00)	27.00 (45.00)	9.00 (45.00)	0.036 ± 0.005	0.045 ± 0.010	0.090 ± 0.013	0.047 ± 0.008	0.097 ± 0.001	0.009 ± 0.001	0.007 ± 0.002	0.047 ± 0.034
^*∗*^Mn	1.20 (2.00)	1.50 (3.00)	2.30 (11.00)	2.30 (11.00)	1.80 (11.00)	1.80 (11.00)	2.00 (11.00)	2.60 (11.00)	0.075 ± 0.019	0.120 ± 0.024	0.373 ± 0.051	0.153 ± 0.048	0.363 ± 0.047	0.043 ± 0.007	0.036 ± 0.010	0.166 ± 0.140

Adapted and modified from Ross et al. [26].

^*∗*^Adequate intake.

Values in brackets: tolerable upper limit.

**Table 5:** Recommended dietary allowances (RDA, mg/day/person) and percent contribution of estimated dietary intake of metal to RDA by consumption of leaf brew and infusion extracts of *L. javanica *(2 g, 5 minutes) collected from seven sampling sites of Marondera, Zimbabwe.

Metal	Mean EDI/RDA percent for life stage group (years) for tea brew								EDI/RDA percent for life stage group (years) for tea infusion							
	Children		Males		Females		Pregnancy	Lactation	Children		Males		Females		Pregnancy	Lactation
	1–3	4–8	19–50	>51	19–50	1–3	4–8	19–50	1–3	4–8	19–50	>51	19–50	>51	19–50	19–50

Cr	2.72	2.00	0.86	1.00	1.20	1.50	1.00	0.67	4.55	3.33	1.43	1.67	2.00	2.5	1.67	1.11
Cu	0.59	0.45	0.22	0.22	0.22	0.22	0.20	0.15	1.18	0.68	0.33	0.33	0.33	0.33	0.30	0.31
Fe	0.52	0.36	0.46	0.46	0.20	0.46	0.13	0.40	0.67	0.47	0.58	0.58	0.26	0.58	0.17	0.52
Mn	9.75	7.80	5.09	5.09	6.50	6.50	5.85	4.50	13.83	11.07	7.22	7.22	9.22	9.22	8.30	4.46
